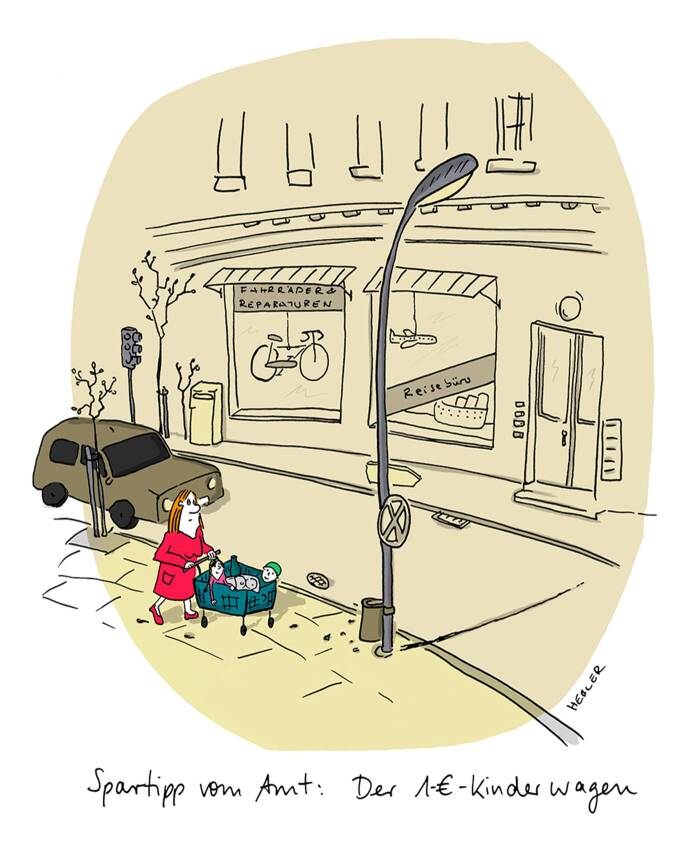# Pole, die nicht schmelzen: Soziale Ungleichheit und Corona

**DOI:** 10.1007/s12054-020-00341-z

**Published:** 2020-12-30

**Authors:** Barbara Lochner, Michael Klundt

**Affiliations:** 1 Fachhochschule Erfurt, Erfurt, Deutschland; 2 Magdeburg, Deutschland

**Keywords:** Soziale Polarisierung, Soziale Ungleichheit, Sozialstaat, Armut, Covid-19

## Abstract

Nicht erst durch Covid-19 sind Kinder und Jugendliche in Armutslagen Benachteiligungen in allen zentralen Lebensbereichen ausgesetzt, die sich u. a. mit sozialer Polarisierung begründen lassen. Der Beitrag leitet in den Schwerpunkt ein, in dem Grundzüge und Zusammenhänge gesellschaftlicher Polarisierungsprozesse beleuchtet und sozialpolitische Ansätze, diesen zu begegnen, diskutiert werden.

Die Zunahme von sozialer Ungleichheit und Prozesse sozialer Polarisierung können in Deutschland seit Jahrzehnten nachgezeichnet werden. Bislang hat dies jedoch kaum dazu geführt, dass wirksame Gegenstrategien implementiert wurden. Die Covid-19-Pandemie wirkt nun wie ein Brennglas und lässt sozial- und bildungspolitische Versäumnisse hinsichtlich der Bekämpfung ungleicher Chancen des Aufwachsens von Kindern und Jugendlichen noch stärker zu Tage treten.

Mittlerweile liegen erste Studienbefunde zu den sozialen Folgen von Covid-19 vor, die deutliche Hinweise darauf geben, dass die pandemiebedingten gesellschaftlichen Einschränkungen im ungleichen Maß zu Belastungen für junge Menschen und Familien führen. So zeigen etwa Andresen u. a., dass Geldsorgen in Folge der Pandemie von der Familienform und „nicht zuletzt von Erwerbsarbeitsverhältnissen“ abhängen (Andresen et al. 2020, S. 22). Die Autor_innen dieser Studie heben die besondere Belastung von Ein-Eltern-Familien hervor (ebd., S. 21). Dass die Sicherheit und Verlässlichkeit von Erwerbsarbeitsverhältnissen Indikatoren dafür sind, wie gut Familien die Situation der Schul- und Kita-Schließungen bewältigen, deutet sich auch in den Ergebnissen der Online-Eltern-Befragung „Thüringer Familien in Zeiten von Corona“ an. In dieser äußern Beamte und deren Kinder insgesamt weniger Belastungen und Sorgen als Personen in anderen Erwerbsarbeitsverhältnissen oder Menschen ohne Arbeit (Lochner und Rompczyk 2020).

Mit Blick auf die sozioökonomische Situation der Familien ziehen die Studie „Kindsein in Zeiten von Corona“ des Deutschen Jugendinstituts (Langmeyer et al. 2020), die Umfrage „Unter Druck“ der Vodafone-Stiftung (2020) wie auch eine Studie der Bertelsmann-Stiftung zum gesellschaftlichen Zusammenhalt in Deutschland (Brand et al. 2020) ein ähnliches Fazit: „In Familien, die mit ihrem gegenwärtigen Haushaltseinkommen kaum zurechtkommen, wird auch die Belastung für die Kinder höher eingeschätzt als in Familien, die ihre finanzielle Lage positiver beurteilen“ (Langmeyer et al. 2020, S. 22; vgl. auch Brand et al. 2020, S. 77). Die Studie der Vodafone-Stiftung widerlegt zudem das Vorurteil, dass Eltern, die selbst nur über einen niedrigen Bildungsabschluss verfügen, sich weniger Gedanken um die schulischen Belange ihrer Kinder machen würden. Im Gegenteil: Während 45 % der Eltern mit einem hohen Bildungsabschluss befürchten, ihre Kinder könnten den schulischen Anschluss verlieren, machen sich 63 % der Eltern mit formal niedriger Bildung entsprechende Sorgen (Vodafone-Stiftung 2020, S. 3). Dass sich die Benachteiligungen von Kindern, die in sozioökonomisch prekären Verhältnissen aufwachsen, 2020 verschärft haben, bilanziert auch eine Studie des Instituts der Deutschen Wirtschaft auf Basis einer Auswertung der SOEP-Daten. Der Autor Geis-Thöne plädiert im Fazit dafür, politische Maßnahmen insbesondere darauf hin zu orientieren, die Situation von Kindern, die von Benachteiligungen betroffen sind, zu verbessern (Geis-Thöne 2020, S. 20). Dies erscheint auch deshalb erforderlich, weil Menschen in sozioökonomisch schwierigen Lebenssituationen auch stärker am gesellschaftlichen Zusammenhalt zweifeln (Brand et al. 2020, S. 43).

Die benannten Studien verweisen insgesamt auf Entwicklungen, die keineswegs erst durch Covid-19 in Gang gesetzt wurden. Vielmehr lassen sich Benachteiligungen von Menschen, die in Armut leben, seit langem nachzeichnen. Armut wirkt sich in komplexer Weise auf alle Lebensbereiche, etwa Bildung, Gesundheit und Wohnen, aus und produziert relational zur gesamtgesellschaftlichen Wirklichkeit Ausschließungserfahrungen (Lochner und Thole 2018). Die Beiträge dieses Schwerpunkts zielen darauf ab, insbesondere die Situation von Kindern in den Blick zu nehmen und Zusammenhänge und strukturelle Ursachen von „polarisierten Kindheiten“ (Hilke & Schütte in diesem Schwerpunkt) sowie Gegenstrategien und Reformkonzepte zu beleuchten.

- Maren Hilke und Johannes Schütte erläutern in ihrem Beitrag gesellschaftliche Polarisierungen in ökonomischer, räumlicher und sozialer Hinsicht. Ihre Ausführungen belegen nicht nur, dass die Ungleichheitsphänomene, die im Zusammenhang mit der Covid-19-Pandemie diskutiert werden, im Kontext langfristiger Polarisierungsprozesse einzuordnen sind. Sie zeigen zudem auf, wie die gesellschaftlichen Ungleichheits- und Machtverhältnisse die Chancen von Kindern von Beginn an in jeglicher Hinsicht prägen.
- Im Anschluss daran fokussiert Michael Klundt aktuelle Entwicklungen. Er betont, dass – entsprechend der These sozialer Polarisierung – keineswegs alle unter den Auswirkungen der Covid-19-Pandemie leiden, sondern es durchaus „Pandemiegewinner“ gibt. Deshalb weist er darauf hin, dass bei allen sozialen Nöten nicht der gestiegene private Reichtum in dieser Gesellschaft vergessenen werden darf. Seiner Analyse zufolge verschärft sich die Differenz in den Lebenswirklichkeiten von Kindern auch deshalb, weil Schutz‑, Fürsorge- und Beteiligungsrechte von Kindern bislang im politischen Pandemie-Management keine oder höchstens eine untergeordnete Rolle spielten.
- Dieser Wahrnehmung schließt sich Christoph Butterwegge an, wenn er betont, dass Kinder und Jugendliche „zu den Hauptleidtragenden der Covid-19-Pandemie“ gehören. Er diskutiert Armut und soziale Ungleichheit als Handlungsauftrag, der politikfeldübergreifend angenommen werden muss. Zentrale Handlungsbedarfe benennt er in den Bereichen der Arbeits- und Beschäftigungspolitik, der Sozial- und Familienpolitik, der Bildungspolitik sowie der Städtebau- und Wohnungspolitik. Lösungsansätze auf der Mikroebene (in den Familien oder in den Einrichtungen des Bildungs- und Sozialwesens) erachtet Butterwegge als unzureichend.
- Jana Liebert widmet sich schließlich der Idee einer Kindergrundsicherung, die seit etwa 15 Jahren als konkrete politische Maßnahme zur Bekämpfung von Kinderarmut diskutiert wird. Mittlerweile liegen verschiedene Modelle der Kindergrundsicherung vor, die die Autorin in ihrem Beitrag anhand ausgewählter Kriterien vergleicht und bewertet. Als entscheidend sieht sie an, in welchem Umfang über die jeweiligen Umsetzungsweisen Teilhabe gewährleistet und Armut reduziert werden kann. Damit verbundene Fragen scheinen bislang noch nicht hinreichend geklärt zu sein, gewinnen aber zunehmend an Brisanz.

Wird den Befunden gefolgt, dass Armut und finanzielle bzw. arbeitsbezogene Unsicherheiten, die Belastungen, die Menschen aufgrund der Covid-19-Pandemie erleben, verstärken, während stabile, sichere und auskömmliche Arbeits- und Einkommensverhältnisse sogar dazu führen können, dass Menschen die Einschränkungen des öffentlichen Lebens als Entspannung empfinden, dann scheinen politische Strategien, die eine verlässliche Existenzsicherung zum Ziel haben, zwingend erforderlich. Darüber hinaus weisen die Beiträge dieses Schwerpunkts aber auch darauf hin, dass sich der gesellschaftliche Zusammenhalt nicht nachhaltig stabilisieren lässt, wenn diese Sicherheit auf einem zunehmend ungleichen Niveau gewährt wird. Covid-19 macht deutlich, dass das was umweltpolitisch eine Katastrophe darstellt, sozialpolitisch dringend angezeigt erscheint: Die Pole müssen wieder schmelzen …